# AKG Attenuates Cerebral Ischemia-Reperfusion Injury through c-Fos/IL-10/Stat3 Signaling Pathway

**DOI:** 10.1155/2022/6839385

**Published:** 2022-05-10

**Authors:** Weilong Hua, Xiaoxi Zhang, Haishuang Tang, Chen Li, Ning Han, He Li, Hongyu Ma, Pei Liu, Yihan Zhou, Hongjian Zhang, Yongxin Zhang, Lei Zhang, Zifu Li, Hongjian Shen, Pengfei Xing, Longjuan Yu, Yongwei Zhang, Yu Zhou, Pengfei Yang, Jianmin Liu

**Affiliations:** ^1^Neurovascular Center, Changhai Hospital, Naval Medical University, Shanghai, China; ^2^Neurovascular Center, Naval Hospital of Eastern Theater, Zhoushan, China

## Abstract

Inflammation is dominant in the pathogenesis of ischemic stroke (IS). Alpha-ketoglutarate (AKG), according to previous studies, has demonstrated a variety of pharmacological effects such as antioxidation and inhibitive inflammation activities. However, whether AKG ameliorates cerebral ischemic injury, as well as the underlying molecular events, is still unclear. Therefore, the effect and underlying mechanisms of AKG on ischemic brain injury should be identified. The study established a cerebral ischemia-reperfusion (I/R) model in mice as well as an oxygen-glucose deprivation/reperfusion (OGD/R) model in SH-SY5Y cells, respectively. It was observed that AKG markedly suppressed infarction volume and neuronal injuries and improved the neurological score in vivo. Moreover, AKG reduced the inflammatory response and lowered the expression of proinflammatory cytokines. In vitro, AKG treatment strongly inhibited OGD/R-induced neuronal injury and the proinflammatory factors. It was also found that the increased SOD and GSH levels, as well as the lower ROS levels, showed that AKG reduced oxidative stress in OGD/R-treated SY-SY5Y cells. Mechanistically, AKG largely promoted IL-10 expression in ischemic brain injury and OGD/R-induced neuronal injury. Furthermore, IL-10 silencing neutralized the protective effect of AKG on inflammation. Notably, it was discovered that AKG could upregulate IL-10 expression by promoting the translocation of c-Fos from the cytoplasm to the nucleus. The results indicated that AKG demonstrated neuroprotection on cerebral ischemia while inhibiting inflammation through c-Fos/IL-10/stat3 pathway.

## 1. Introduction

Cerebral ischemia/reperfusion serves as the third dominating factor of disability and mortality worldwide [1, 2]. It is evidently suggested that the pathogenesis of ischemic stroke is extremely complex. Inflammation, autophagy, excitotoxicity, apoptosis, and oxidative stress are indispensable in the pathophysiology of cerebral ischemia/reperfusion damage [3, 4]. Although clinical management of ischemic brain damage has been studied at multiple levels, feasible drug therapies for reversing the progression of ischemia/reperfusion injury are scarce [5]. Therefore, further exploration and development of new drugs are urgently needed for IS.

The pathophysiological mechanism of ischemic brain damage is associated with inflammatory response. The inflammatory response contributes to secondary neuronal damage and a substantial detrimental impact on the advancement of tissue injury [6]. After ischemic injury, several cytokines increased in neurons and glial cells, as well as the cells of the immune system [7]. Although some cytokines aggravate cerebral damage, other cytokines, such as interleukin-10 (IL-10), a neurotrophic cytokine containing neurons and glia, exert a neuroprotective role during stroke [8]. Published work validated the protective mechanism of IL-10 towards cortical neurons via the activation of PI-3 kinase and STAT-3 pathways [9]. Preexisting interleukin-10 in cerebral arteries showed mitigation of brain damage induced by ischemic brain injury following MCAO [10]. The experiment suggested that IL-10 may function as a neuroprotective agent during cerebral ischemia/reperfusion.

Alpha-ketoglutarate (AKG) is an essential intermediary in the tricarboxylic acid cycle between succinyl-CoA and isocitrate [11]. AKG serves in ATP production through mitochondrial oxidative phosphorylation and regulates immune system homeostasis and ROS level [12]. Recent studies claimed that dietary AKG supplementation reduced the reactive oxygen species [13], boosting a longer and healthier life with less systemic inflammatory cytokines [14]. According to the results, AKG may protect against ischemic stroke with inflammation and reactive oxygen species. Nevertheless, data supporting this hypothesis have been largely lacking. Attempts are required to test the hypothesis regarding the protective role of AKG in cerebral IS.

The neuroprotective impact of AKG on the brain of mice following ischemia-reperfusion injury was studied as well as neuronal injury in cultured SH-SY5Y cells following OGD/R. The neuroprotective mechanisms and targets of AKG in cerebral I/R injury were elucidated. More importantly, AKG plays a neuroprotective role through the c-Fos/IL-10 pathway.

## 2. Materials and Methods

### 2.1. Animal Study

The Animal Care and Use Committee of Changhai Hospital Affiliated to Naval Medical University granted their approval to the experiments. All of the mice, housed in a controlled setting with humidity, temperature, and light cycles, were provided with unrestricted supply of food and water.

Fourteen days before MCAO, mice were randomly assigned to receive either drinking water or AKG (Macklin, China) dissolved in drinking water. The procedure for occlusion of the middle cerebral artery (MCAO) was conducted as reported previously [15]. Male mice were sedated thoroughly with 1 percent pentobarbital. The left common carotid artery was exposed. Both the internal and exterior carotid arteries were properly dissected. The external carotid artery was ligatured with a 5-0 silk suture. The internal carotid artery (ICA) was then entered with a 7–0 surgical nylon monofilament with a rounded end, obstructing the middle cerebral artery (MCA) for 90 minutes. The suture was then withdrawn to enable blood flow, while the incision and skin were closed.

Mice were given either drinking water or AKG for 24 hours after reperfusion. The neurological deficiency score was then calculated. The neurological dysfunction in mice was evaluated in the same method stated above [16].

### 2.2. Histological Examination, TUNEL Staining, and Nissl Staining

H&E was applied to observe the abnormal indications in the brain tissue. Paraffin brain slices were cut at 5 m, stained with HE (eosin, hematoxylin), viewed with a fluorescence microscope for histological study (Nikon, Japan). The TUNEL assay was performed suing a Roche TUNEL kit in accordance with careful instructions. A fluorescent microscope acquired the image of TUNEL-positive neurons surrounding the damaged sites (Olympus, Japan).

Paraffin slices underwent a five-minute dewaxing with xylene and dyed with Nissl solution. After that, the slices were soaked in 95% alcohol until the Nissl bodies turned dark blue and the backdrop turned colorless or light blue. The microscope displayed the Nissl bodies in cortical neurons.

### 2.3. Triphenyltetrazolium Chloride (TTC) Staining

Two-millimeter coronal segments of isolated brain tissues were then fixed in 4 percent formaldehyde at 4°C for 24 hours under 2% TTC immersion (Sigma-Aldrich, United States) for 30 minutes at 37°C in the dark. The ImageJ software enabled the calculation of the infarct volume, expressed as a proportion of the brain tissue.

### 2.4. Cell Culture and Oxygen-Glucose Deprivation/Reoxygenation (OGD/R) Model

The SH-SY5Y cell line was grown in DMEM/F12 media added 10% fetal bovine serum and penicillin/streptomycin and incubated at 37°C with 5 percent CO_2_.

The SH-SY5Y cells were washed three times in PBS and pretreated with AKG (10, 50 mmol/L) or stattic (10 *μ*mol/L, Macklin, Shanghai) for one hour before OGD. A glucose-free DMEM medium was used in place of the usual culture media for OGD therapy, with 4-hour hypoxic cells at 37°C with 95 percent N_2_ and 5 percent CO_2_. The culture base for SH-SY5Y cells was the normal media at 37° C for twelve hours for reperfusion, using a normal normoxia in check.

### 2.5. ELISA Assay

SY-SY5Y cells or mouse brain tissue was homogenized with PBS at 4°C and centrifuged at 14,000 rpm. Then, the detection of level of interleukin-10 (IL-10), interleukin-1*β* (IL-1*β*), interleukin-6 (IL-6), and tumor necrosis factor-*α* (TNF-*α*) was conducted with ELISA kits (Abcam) strictly following instructions.

### 2.6. Intracellular Reactive Oxygen Species (ROS) Detection

Applying commercial assay kits, the activities of malondialdehyde, superoxide dismutase, and glutathione in the cells and brain tissues were identified. DCFH-DA fluorescent probes were applied to measure intracellular ROS levels in cells, and DHE fluorescent probes were utilized to evaluate ROS levels in brain tissues. In a light-protected chamber with humidity, the collected samples were incubated using DCFH-DA or DHE for 45 minutes at 37°C. A fluorescent microscope was used to examine the results (Carl Zeiss, Germany).

### 2.7. Real-Time Quantitative Polymerase Chain Reaction (RT-qPCR)

TRIzol reagent was used to isolate total RNA from SY-SY5Y cells or mouse brain tissue at the fringe area of the infarct. SYBR Green was used in the quantitative real-time PCR (Takara, Japan). By normalizing the samples to GAPDH, an internal reference, the samples were relatively measured. Supplementary Table S1 lists the real-time PCR primers applied in this investigation.

### 2.8. Western Blot Assay

RIPA lysis buffer with phosphatase and protease inhibitors was applied to lyse the collected cell samples and tissues. The quantities of protein were then evaluated using a BCA protein assay kit. Electrophoresis of sodium dodecyl sulfate-polyacrylamide gels was utilized to segregate the protein on a polyvinylidene difluoride (PVDF) membrane (SDS-PAGE). Before incubation with fundamental antibodies overnight at 4 degree Celsius, the blots were inhibited for 1 hour at temperate room temperature with 5% nonfat milk. Using an ECL method and secondary antibodies against goat anti-rabbit IgG or goat anti-mouse IgG, antigen-antibody complexes were detected. The protein bands were normalized using GAPDH.

### 2.9. Statistical Analysis

The information is given as a mean with a standard deviation (SEM). The differences between two groups were investigated using the Student's *t*-test. The analysis of variance was aimed at comparing the results of multiple groups (ANOVA). A *P* value < 0.05 was considered as the statistical significance.

## 3. Results

### 3.1. AKG Ameliorated Neurological Deficits and Reduced Infarct Volumes in MCAO Mice

**Figure 1:**
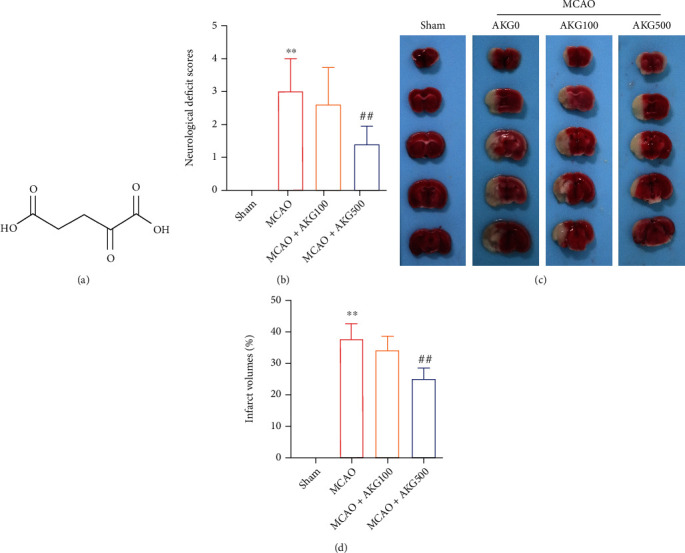
AKG therapy enhanced neurological effects and reduced infarct volumes in MCAO mice. (a) AKG's molecular structure. (b) Effects of AKG on neurological function in mice following cerebral I/R (*n* = 6). (c) TTC staining was employed to approach the area of brain infarction in mice. (d) ImageJ analysis system was used to examine the infarct regions (*n* = 6). ^∗∗^*P* < 0.01 versus the sham group and ^##^*P* < 0.01 versus the MCAO group.

The molecular structure of AKG was shown in Figure 1(a). The MCAO model was applied in mice to determine the effect of AKG. AKG was administered to mice at 100 and 500 mg/kg. Neurological scoring results exhibited that 24 hours after, the MCAO model was established; AKG dramatically reduced the neurological score of mice (Figure 1(b)). In addition, TTC staining revealed a cerebral infarction (Figure 1(c)). TTC staining results showed the reduction of cerebral infarction by AKG (Figure 1(d)).

### 3.2. AKG Reduced Nerve Injury and Cell Apoptosis of the Brain in MCAO Mice

**Figure 2:**
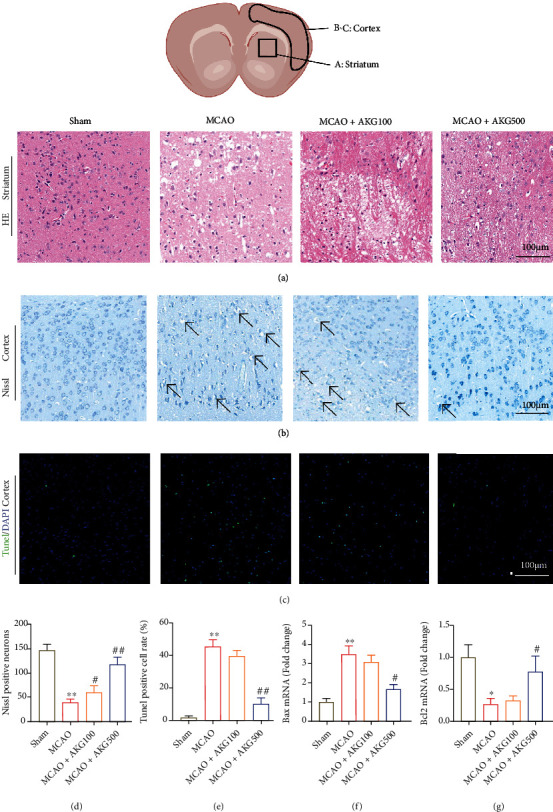
AKG therapy reduced nerve injury and brain cell death in MCAO mice. (a) Hematoxylin and eosin staining experiment (*n* = 7). Scale bar, 100 *μ*m. (b) Nissl staining assay (*n* = 7). Scale bar, 100 *μ*m. (c) TUNEL assay (*n* = 7) (TUNEL, green; DAPI, blue) (*n* = 7). Scale bar, 100 *μ*m. (d and e) The number of Nissl-positive neurons and TUNEL-positive cells was calculated. (f and g) BAX and BCL-2 mRNA levels were determined by RT-qPCR (*n* = 5). ^∗^*P* < 0.05 and ^∗∗^*P* < 0.01 versus the sham group and ^#^*P* < 0.05 and ^##^*P* < 0.01 versus the MCAO group.

The effects of AKG on cell damage in mice brain tissues following MCAO were then investigated. After I/R, a high number of neurons in the cerebral cortex's ischemic penumbra were injured, as demonstrated by HE staining experiments. AKG treatment, on the other hand, greatly improved these outcomes (Figure 2(a)). The injured neurons have shrunken cell bodies with shrunken and pyknotic nuclei and greater dark staining in MCAO group. AKG therapy dramatically enhanced the quantity and shape of nerve cells, as seen in Figures 2(b) and 2(d). The apoptosis of brain tissues was detected by TUNEL labeling. Neuronal apoptosis showed growth in the MCAO group compared to the sham surgery group, but lowered in the AKG therapy group, as seen in Figures 2(c) and 2(e). AKG also prevented the MCAO-induced increases in the expression of BAX while increasing the antiapoptotic protein BCL-2, according to RT-qPCR (Figures 2(f) and 2(g)). Taken together, these findings show that AKG dramatically reduced nerve damage and brain cell death in MCAO animals.

### 3.3. AKG Significantly Inhibited the Inflammatory Response and Oxidative Damage in MCAO Mice

**Figure 3:**
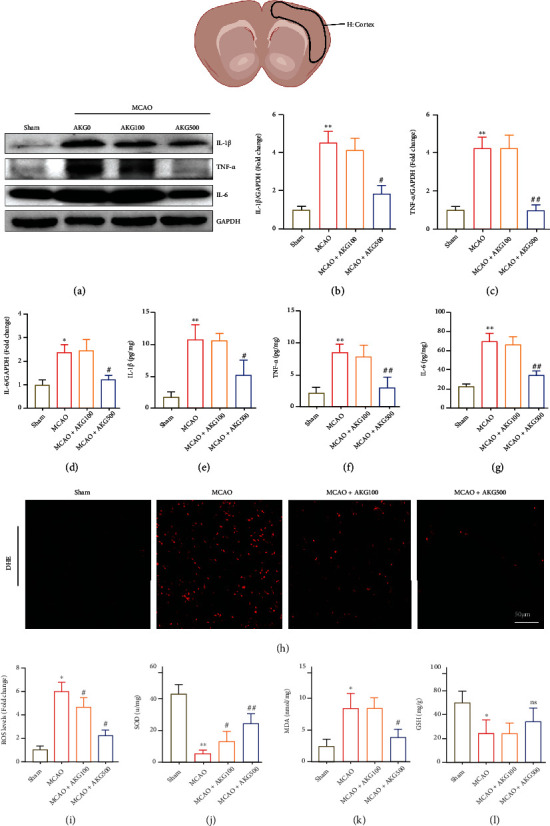
In MCAO mice, AKG therapy decreased inflammatory response and oxidative damage. (a–d) Representative blots of proinflammatory cytokines and quantitative analysis (*n* = 4). (e–g) The levels of proinflammatory cytokines were measured using ELISA (*n* = 5). (h and i) The levels of superoxide anions as determined by DHE staining are shown in representative photos and quantitative analysis (*n* = 5). Scale bar, 100 *μ*m. (j–l) SOD, MDA, and GSH levels were measured using SOD, MDA, and GSH kits (*n* = 5). ^∗^*P* < 0.05 and ^∗∗^*P* < 0.01 versus the sham group and ^#^*P* < 0.05 and ^##^*P* < 0.01 versus the MCAO group.

The expression of proinflammatory cytokines was assessed by western blot after 24 hours of reperfusion. The results revealed that MCAO caused significant elevation of proinflammatory cytokines, which was significantly reduced by AKG therapy (Figures 3(a)–3(d)). The enzyme-linked immunosorbent assay (ELISA) produced similar results (Figures 3(e)–3(g)). AKG therapy also decreased MCAO-induced ROS production, seen in DHE staining (Figures 3(h) and 3(i)). In addition, MDA levels as well as the activities of SOD and GSH were identified, that is, the two important antioxidant enzymes involved in oxidative stress protection in nerve tissue. Presented in Figures 3(j)–3(l), AKG reduced the downregulation of antioxidant activities in MCAO mice, such as GSH, SOD, and MDA, in a dose-dependent manner.

### 3.4. AKG Suppressed OGD/R-Induced Inflammatory Response, Oxidative Stress, and Apoptosis in SH-SY5Y Cells

**Figure 4:**
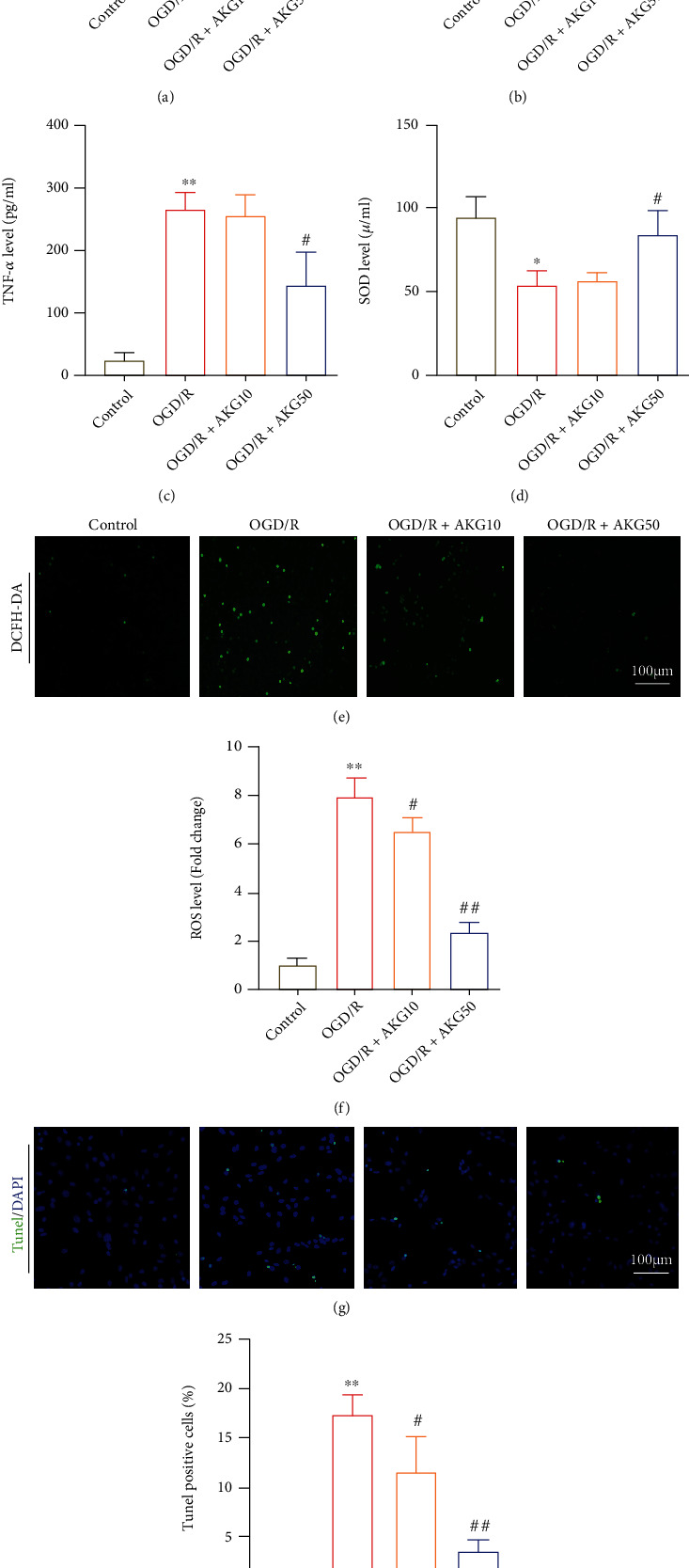
AKG inhibited the inflammatory response, oxidative stress, and apoptosis induced by OGD/R in SH-SY5Y cells. (a–c) The levels of proinflammatory cytokines were measured using ELISA (*n* = 5). (d) The levels of SOD were determined using a SOD kit (*n* = 5). (e and f) The levels of superoxide anions as determined by DCFH-DA staining are shown in representative photos and quantitative analysis (*n* = 5). (g and h) Quantification of TUNEL-positive cells in SH-SY5Y cells in the indicated groups (*n* = 5) (TUNEL, green; DAPI, blue). ^∗^*P* < 0.05 and ^∗∗^*P* < 0.01 versus the control group and ^#^*P* < 0.05 and ^##^*P* < 0.01 versus the OGD/R group.

Ischemia-reperfusion damage is linked to oxidative stress impairment, an inflammatory response, and apoptosis. The levels of proinflammatory factors were assessed in SH-SY5Y cells. In OGD/R-induced SH-SY5Y cells, the levels of IL-1*β*, TNF-*α*, and IL-6 were dramatically raised, as shown in Figures 4(a)–4(c), and AKG completely reversed these alterations. By assessing intracellular ROS generation, the oxidative stress in OGD/R-induced SH-SY5Y cells associated with AKG was examined. AKG prevented the downregulation of SOD antioxidant activities following OGD/R stimulation, as indicated in Figure 4(d). AKG therapy also decreased OGD/R-induced ROS production, as measured by DHE staining (Figures 4(e) and 4(f)). TUNEL labeling was used to assess the importance of AKG on OGD/R-induced apoptosis in SH-SY5Y cells. The outcome demonstrated that AKG largely reduced the amount of OGD/R-induced TUNEL-positive cells (Figures 4(g) and 4(h)). These findings suggested that AKG could reduce oxidative stress, apoptosis, and inflammation generated by OGD/R.

### 3.5. AKG Targeted IL-10 to Inhibit Cerebral Ischemia-Reperfusion Injury in Mice

**Figure 5:**
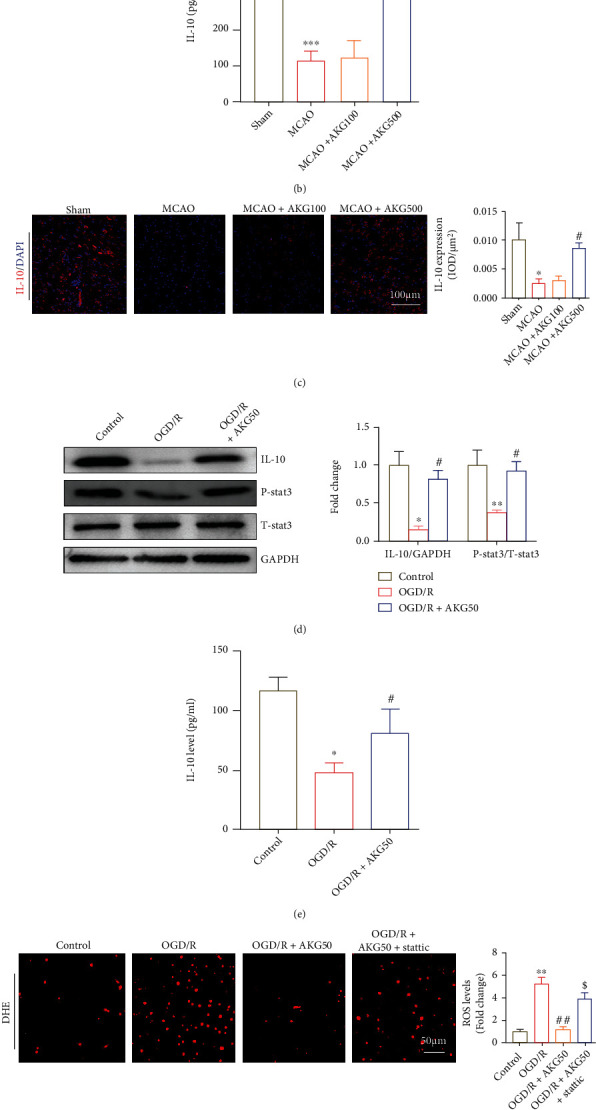
The activation of IL-10/stat3 signaling was linked to the protective effect of AKG against I/R damage. (a) Western blot analysis and quantification of IL-10 and stat3 expression in the indicated groups (*n* = 4). (b) IL-10 levels were measured using an ELISA kit (*n* = 5). (c) Immunofluorescence staining (*n* = 5) (IL-10, red; DAPI, blue). (d) Western blot analysis and quantification of IL-10 and stat3 expression in the indicated groups (*n* = 4). (e) IL-10 levels were measured using an ELISA kit (*n* = 5). (f) The levels of superoxide anions as determined by DHE staining are shown in representative photos and quantitative analysis (*n* = 5). (g) The levels of proinflammatory cytokines were measured using ELISA (*n* = 5). (a–c) ^∗^*P* < 0.05, ^∗∗^*P* < 0.01, and ^∗∗∗^*P* < 0.01 versus the sham group and ^#^*P* < 0.05, ^##^*P* < 0.01, and ^###^*P* < 0.01 versus the MCAO group; (d–g) ^∗^*P* < 0.05 and ^∗∗^*P* < 0.01 versus the control group, ^#^*P* < 0.05 and ^##^*P* < 0.01 versus the OGD/R group, and ^$^*P* < 0.05 and ^$$^*P* < 0.01 versus the OGD/R+AKG50 group.

Previous research has demonstrated that mice produce considerably more IL-10 and have a longer lifetime than untreated animals under AKG [14]. IL-10 serves to maintain the immune system in control. It was speculated that AKG suppressed cerebral ischemia-reperfusion injury and oxidative stress by promoting IL-10 expression. Western blotting and ELISA were applied in vivo to confirm the altered expression of IL-10, which was consistent with earlier findings (Figures 5(a) and 5(b)). Similar results were also observed by immunofluorescence staining (Figure 5(c)). Next, we detect the IL-10 expression in vitro. AKG promoted the expression of IL-10 expression in SH-SY5Y cells, according to the findings (Figures 5(d) and 5(e)). The IL-10/stat3 axis has been linked to brain damage in the previous research [17]. Western blot examination consistently revealed that AKG therapy promoted the expression of p-stat3 as compared to the MCAO group (Figure 5(a)). In vitro investigations also yielded similar results (Figure 5(d)). Furthermore, stattic, the employment of a stat3 inhibitor, attenuated the protective effect of AKG. Compared to AKG treatment group, stattic increased the ROS generation, as shown by DHE staining (Figure 5(f)). ELISA revealed that stattic inhibited AKG-mediated negative regulation of the levels of IL-1*β*, TNF-*α*, and IL-6 (Figure 5(g)). Collectively, these results suggested that AKG suppressed OGD/R-induced inflammatory response and oxidative stress through IL-10/stat3 pathway.

### 3.6. A Knockdown of IL-10 with Small Interfering RNA (siRNA) Exacerbates Cerebral Ischemia-Reperfusion Injury In Vitro

**Figure 6:**
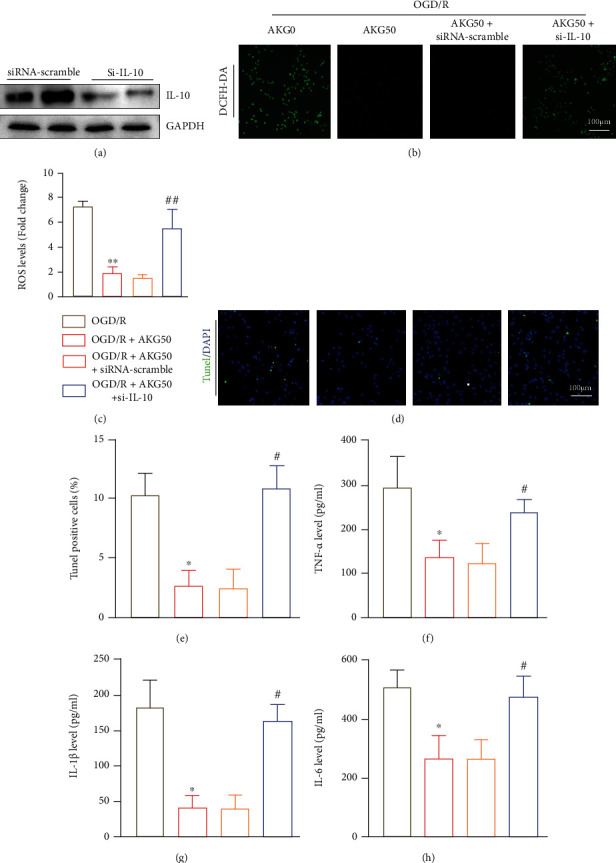
In vitro, suppressing IL-10 greatly reduced the protective effect of AKG. (a) Western blot analysis of IL-10 expression (*n* = 4). (b and c) Representative pictures and quantitative analysis of superoxide anions levels as evaluated by DCFH-DA staining (*n* = 5). Scale bar, 100 *μ*m. (d and e) Quantification of TUNEL-positive cells in SH-SY5Y cells in the indicated groups (*n* = 5) (TUNEL, green; DAPI, blue). Scale bar, 100 *μ*m. (f–h) The proinflammatory cytokines were measured using ELISA (*n* = 5). ^∗^*P* < 0.05 versus the OGD/R group and ^#^*P* < 0.05 versus the OGD/R+AKG50 group.

The IL-10-specific siRNAs were designed to transfect SH-SY5Y cells followed by OGD/R stimulation for 24 hours to further elucidate the impact of IL-10 in AKG-mediated modulation of SH-SY5Y cells. Western blotting confirmed the effectiveness of the IL-10 knockdown (Figure 6(a)). IL-10-siRNA significantly reduced the inhibitory effect of AKG on ROS production (Figures 6(b) and 6(c)), apoptosis (Figures 6(d) and 6(e)), and inflammatory response (Figures 6(f)–6(h)). Overall, it was revealed that suppressing IL-10 in vitro severely decreased the protectiveness of AKG.

### 3.7. AKG Promoted IL-10 Expression by Enhancing AP-1 Activity in SH-SY5Y Cells

**Figure 7:**
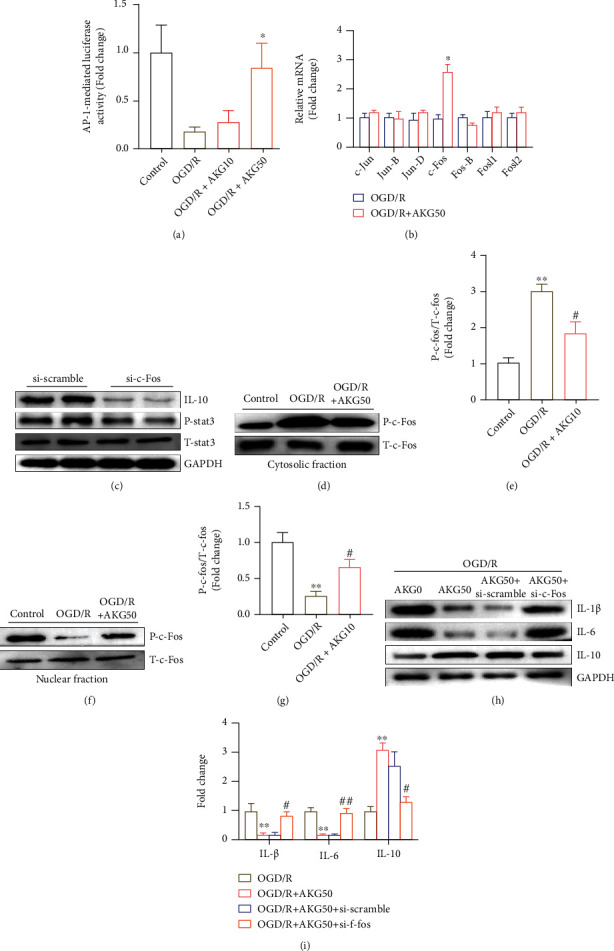
AKG promoted the activity and expression of c-Fos, which increased OGD/R-induced IL-10 expression. (a) An AP-1 transcription factor assay kit was used to evaluate AP-1 activity (*n* = 5). (b) Jund, Junb, c-Jun, Fos-b, c-Fos, Fosl2, and Fosl1 mRNA levels in different treatments by using RT-qPCR (*n* = 4). (c) Representative blots and quantitative analysis of IL-10 and stat3 (*n* = 4). (d–i) Representative blots and quantitative analysis of P-c-Fos, T-c-Fos, IL-1, IL-6, and IL-10 (*n* = 4 per group). (a and b) ^∗^*P* < 0.05 versus the OGD/R group. (e and g) ^∗∗^*P* < 0.01 versus the OGD/R group. (i) ^∗∗^*P* < 0.01 versus the OGD/R group and ^#^*P* < 0.05 and ^##^*P* < 0.01 versus the OGD/R+AKG50 group.

Activator protein 1 is a transcription factor complex that can transactivate IL-10 [18, 19]. It is made up of the Fos and Jun families. As a result, it was hypothesized that the protection of AKG against cerebral ischemia-reperfusion injury was linked to the AP-1 complex. We found that AKG facilitated the AP-1 activity (Figure 7(a)). RT-qPCR analysis pointed out that c-Fos expression was higher with AKG than the OGD/R group (Figure 7(b)). It was discovered that downregulating c-Fos levels decreased the expression of IL-10 and P-stat3 in SH-SY5Y cells after transfecting them with si-c-Fos (Figure 7(c)). Additionally, P-c-Fos protein distribution in nucleus was observed. In SH-SY5Y cells, Western blotting revealed that AKG significantly aided the translocation of p-c-Jun from the cytoplasm to the nucleus (Figures 7(d)–7(g)). In the different treatment groups, c-Fos silencing boosted the IL-1*β* and IL-6 while decreasing the IL-10 (Figures 7(h) and 7(i)). In conclusion, these findings showed that AKG promoted IL-10 expression by inhibiting AP-1 activity in SH-SY5Y cells.

## 4. Discussion

This study showed that AKG demonstrate a neuroprotective role in cerebral ischemia by promoting IL-10 protein. In vivo, AKG supplementation significantly ameliorated neurological deficits, infarct volumes, nerve injury, and cell apoptosis of the brain in MCAO mice. In vitro, AKG treatment markedly suppressed OGD/R-induced neuronal in SH-SY5Y cells. Furthermore, the protective AKG against cerebral I/R injury was associated with the IL-10/stat3. Regarding IL-10 silencing in vitro, the protective effect of AKG was dramatically reduced. Importantly, AKG protects mice from ischemia-reperfusion damage by c-Fos/IL-10/Stat3 signaling pathway.

The cerebral I/R injury is a complicated disease that causes the number of fatality and disability, accompanied by cognitive dysfunctions [20]. Previous research has linked ischemic stroke to increasing reactive oxygen species (ROS) [21], excitotoxicity, acidotoxicity, ionic imbalance, and excitotoxicity [22], and inflammation [23], which are important contributors to secondary brain damage after stroke. However, treatment of cerebral ischemia remains a clinical challenge for researchers. Recent studies indicate that AKG supplementation, a major molecule in the TCA cycle, reduced the production of ROS in mouse experiment [24]. The significant effects of AKG on oxidative stress and inflammation imply that AKG could be used to treat ischemic stroke. AKG reduced the downregulation of antioxidant activities in MCAO mice, including GSH, MDA, and SOD. AKG also decreased the proinflammatory cytokines in vivo as well as in vitro, according to our findings. These findings were in line with earlier research in a variety of cell types and organisms. AKG decreases chronic inflammation and promotes IL-10 in female mouse T cells, according to new research. IL-10 mutant mice have higher levels of proinflammatory cytokines including IL-6, IL-12, TNF-*α*, CXCL-1, and IL-1*β* [14]. IL-10 is a strong inhibiting inflammatory cytokine that helps to keep the immune system in check [25]. Pérez-de Puig et al. found that IL-10 loss increased infarct volume and neurologic impairments marginally [8]. Piepke et al.'s study showed that intracerebral treatment with recombinant IL-10 effectively inhibited cerebral I/R injury [26]. Consistent with these findings, we showed that AKG suppressed cerebral I/R injury by promoting IL-10 expression. Inhibition of IL-10 largely prevented the inhibitory effect of AKG on OGD/R-induced SH-SY5Y cells inflammatory response. Therefore, it was speculated that AKG-induced IL-10 upregulation played the essential protective role in regulating inflammatory response. Previous literature proved that the inhibiting inflammatory response is mediated by the IL-10/STAT3 axis [27, 28]. In this work, lower stat3 phosphorylated protein levels were discovered regarding I/R injury, and these declines were effectively controlled by AKG supplementation.

The AP-1, a transcription factor complex that has been shown to transactivate the IL-10 gene, has been extensively explored [29]. It is made up of members of the Fos and Jun families. The protective role of AKG on cerebral I/R injury was thought to be linked to AP-1 complex activity. This research aimed to the first presenter of the protection of AKG against cerebral ischemia by inhibiting inflammation via the c-Fos/IL-10/stat3 pathway. AKG treatment increased translocation and expression of p-c-Fos to facilitate IL-10 transcription.

The study showed limitations. First, incomprehensive silence experiments were carried out. Experiments with overexpression should be used to learn more about the mechanism that triggers protectiveness of AKG. Second, in both in vivo and in vitro research, only two dosages were found. Additional studies is required to determine the optimal dose of AKG by looking at its tissue distribution, concentration, and pharmacokinetics. Third, there is insufficient clinical to directly confirm the relationship between AKG and cerebral I/R disease. The changes of circulating AKG content in cerebral I/R disease need to be further explored.

These findings were the first to show that AKG inhibited the inflammatory response and oxidative damage in the brain during ischemia-reperfusion injury. Notably, we discovered that AKG could reduce cerebral I/R injury at least partly with c-Fos/IL-10/stat3 signaling pathway. AKG has potential as a treatment for cerebral I/R injury.

## Data Availability

The data that support the findings of this study are available from the corresponding author upon reasonable request.

## Supplementary Materials

Supplementary Materials Table S1: list of RT-PCR primers.
