# Supplementary material for: AKG Attenuates Cerebral Ischemia-Reperfusion Injury through c-Fos/IL-10/Stat3 Signaling Pathway
Source: Oxid Med Cell Longev. 2022 May 10;2022:6839385. doi: 10.1155/2022/6839385 (PMC9113869; doi:10.1155/2022/6839385)
Supplement: Supplementary Materials — Table S1: list of RT-PCR primers. [file 6839385.f1.zip › Supplementary Description.docx]

Supplementary Description:

Table S1. List of RT-PCR primers
