# Supplementary material for: AKG Attenuates Cerebral Ischemia-Reperfusion Injury through c-Fos/IL-10/Stat3 Signaling Pathway
Source: Oxid Med Cell Longev. 2022 May 10;2022:6839385. doi: 10.1155/2022/6839385 (PMC9113869; doi:10.1155/2022/6839385)
Supplement: Supplementary Materials — Table S1: list of RT-PCR primers. [file 6839385.f1.zip › Table S1. List of RT-PCR primers.pdf]

Table S1. List of RT-PCR primers

Genus	mRNA	Forward	Reverse
Mouse	Bcl2	GTCGCTACCGTCGTGACTTC	CAGACATGCACCTACCCAGC
Mouse	Bax	TGAAGACAGGGGCCTTTTTG	AATTCGCCGGAGACACTCG
Human	JUN	TCCAAGTGCCGAAAAAGGAAG	CGAGTTCTGAGCTTTCAAGGT
Human	JUNB	ACGACTCATACACAGCTACGG	GCTCGGTTTCAGGAGTTTGTAGT
Human	JUND	TCATCATCCAGTCCAACGGG	TTCTGCTTGTGTAAATCCTCCAG
Human	FOS	CCGGGGATAGCCTCTCTTACT	CCAGGTCCGTGCAGAAGTC
Human	FOSB	GCTGCAAGATCCCCTACGAAG	ACGAAGAAGTGTACGAAGGGTT
Human	FOSL1	CAGGCGGAGACTGACAAACTG	TCCTTCCGGGATTTTGCAGAT
Human	FOSL2	CAGAAATTCCGGGTAGATATGCC	GGTATGGGTTGGACATGGAGG
